# Synergy Analysis Between the Temporal Dominance of Sensations and Temporal Liking Curves of Strawberries

**DOI:** 10.3390/foods14060992

**Published:** 2025-03-14

**Authors:** Shogo Okamoto, Hiroharu Natsume, Hiroki Watanabe

**Affiliations:** Department of Computer Science, Tokyo Metropolitan University, Tokyo 192-0397, Japan; natsume-hiroharu@ed.tmu.ac.jp (H.N.); watanabe-hiroki8@ed.tmu.ac.jp (H.W.)

**Keywords:** non-negative matrix factorization, supervised learning, strawberry, principal motion

## Abstract

The Temporal Dominance of Sensations (TDS) method allows for the real-time tracking of changes in multiple sensory attributes, such as taste, aroma, and texture, during food tasting. Over the past decade, it has become an essential tool in sensory evaluation, offering novel insights into temporal sensory perception. When combined with the Temporal Liking (TL) method, TDS enables the investigation of how sensory changes influence instantaneous liking. Existing methods in time-series sensory evaluation have not simultaneously achieved the following two key objectives: (1) predicting TL curves from TDS curves and (2) identifying shared sensory–liking synergies across samples. In this study, we address this gap by applying supervised non-negative matrix factorization, which enables both precise prediction and interpretable synergy extraction. This novel approach has the potential to extend the applicability of TDS analysis to broader sensory evaluation contexts. We validated the method using the data for strawberries recorded in an earlier study. Our model, utilizing three latent synergy components accounting for 94% of the data variation, accurately predicted the TL curves from TDS curves with a median RMSE of 0.36 in cross-validation, approximately 1/16 of the maximum TL score. Moreover, these synergy components were highly interpretable, suggesting some key factors that explain individual variations in sensory perception. These findings highlight the effectiveness of synergy analysis in time-series sensory evaluation, leading to deeper understanding of the connections between temporal sensory and liking responses.

## 1. Introduction

The Temporal Dominance of Sensations (TDS) method [1,2,3] has become an essential tool in the field of food science over the past decade. This method allows for the simultaneous recording of temporal changes in taste, aroma, and texture while tasting food, providing valuable information for food developers and researchers. The TDS method is widely applied for food brand classification and the visualization of dynamic tasting experiences [4,5,6,7,8,9,10]. The TDS is often used in combination with the Temporal Liking (TL) method [11,12,13,14,15,16,17], enabling the analysis of how dynamically changing sensory attributes influence the instantaneous liking of a food product. For instance, Thomas et al. [11] examined the average TL score during the period when a specific sensory attribute was dominant and discussed temporal drivers of liking—the key attributes influencing liking over time. Establishing a link between dynamically changing sensory attributes and liking is one of the primary objectives of using TDS methods [18,19,20].

A distinguishing feature of TDS methods is their ability to capture multivariate temporal changes. However, interpreting the complex temporal information embedded in TDS curves (time-evolving profiles of dominant sensations) can be challenging. Various methods have been developed to aid in the interpretation of TDS data. For example, differential curves [1] effectively visualize differences between two food brands, while trajectory plots [8,9,21] are useful for visually comparing more than three brands within a principal component space. Additionally, analysis techniques such as state transition diagrams [22,23] and causal networks [24] have been proposed to illustrate dominant sensory transitions.

When TDS and TL methods are used together, identifying interdependent components between the two sets of temporal curves becomes even more challenging. To date, no study has explicitly attempted to quantify the synergy between TDS and TL curves.

A potentially effective approach to addressing this challenge is synergy analysis, a method commonly used to identify coordinated patterns in systems with high degrees of freedom and interdependent components, such as human limb and muscle movements [25,26,27,28,29,30,31,32,33,34]. Synergy analysis is particularly useful for discovering multivariate temporal coordination and extracting underlying bases governing interdependent variables. For example, Mishima et al. [30] identified dominant coordination patterns among 14 inertial measurement units placed on the human body during walking and lifting tasks. Similarly, Qiu et al. [32] analyzed multiple sit-to-stand motions under varying handrail and foot positions to extract key motion patterns that influence perceived ease of standing up.

To our knowledge, only one study has applied synergy analysis to TDS curves [5]. In that study, three principal coordinated motions were extracted from TDS curves obtained for multiple brands of umeboshi (pickled plums). Each motion represented a distinct sensory synergy, such as sourness and saltiness, sweetness and smooth texture, and umami and sourness. Based on these principal motions, different brands were successfully classified, and their characteristic taste profiles were clearly defined. However, this study did not explore the synergy between TDS and TL curves.

Recently, researchers have begun investigating methods to predict TL curves from TDS curves [17,35]. Beyond providing a mathematical framework for modeling the relationship between TDS and TL, such techniques have the potential to reduce the experimental cost of TL curve collection in the future. For example, Natsume and Okamoto [35] applied reservoir computing, a type of recurrent neural network, to predict the TL curve of an instant coffee brand using TDS and TL curves from three other coffee brands. The prediction of TL curves from TDS curves is still in its early stages, and the methodology remains underdeveloped.

This study addresses the following two key challenges related to TDS and TL curves: (1) extracting principal synergies between TDS and TL curves, and (2) predicting TL curves from TDS curves.

As discussed earlier, research on these individual problems remains limited, and no existing analytical method has been applied to simultaneously solve both challenges within the domain of time-series sensory evaluation. In this study, we propose applying supervised non-negative matrix factorization as a potential solution to these problems. To evaluate the effectiveness of this approach, we apply it to the TDS and TL curves of strawberries obtained in a previous study [17], assess its performance, and discuss its limitations and future directions.

## 2. Materials and Methods

We utilize the strawberry dataset obtained using the TDS and TL methods from a previous study [17]. Details can be found in that study. In this paper, we provide a summary of the TDS and TL tasks along with example data to aid comprehension. Notably, this study does not involve any new experiments.

### 2.1. TDS and TL Tasks for Strawberries

In the previous study [17], tasting experiences of strawberries (Tochi-Aika cultivar) were collected using the TDS and TL methods. The study involved thirty-one university students and was conducted with the approval of the institutional review board of Hino Campus, Tokyo Metropolitan University (Approval number: R6-008). All participants performed each of the TDS and TL tasks three times in a counterbalanced manner. A tailored software application developed in Python 3 (version 3.12.3) was used as the computer interface for these tasks.

The TDS task recorded the following eight types of sensory attributes:

- Aromatic: complex and pleasant smell.
- Juicy: perception of juice and flesh content.
- Sweet: basic sweet taste.
- Fruity: smell of sweet fruits.
- Light: sweet taste that does not linger in the mouth.
- Watery: perception of water content without strong taste.
- Green: smell, taste, and mouthfeel of grass or unripe fruits.
- Sour: basic sour taste.

These attributes were originally selected by Shimaoka et al. [36] to comprehensively represent the dominant sensory properties of strawberries in TDS tasks. At each moment, only one of these attributes was selected in accordance with the standard TDS method [2]. Each participant consumed a whole strawberry in a single bite for each trial. They started and ended the recording themselves from when they placed the food sample in their mouth and until it was completely swallowed.

The TL task recorded the degree of instantaneous liking while tasting strawberries. This rating was on a 9-point scale (1: least; 9: most), and the participants pressed one of the nine buttons whenever their liking changed. Similar to the TDS task, each trial involved consuming a single strawberry piece, and the participant controlled both the start and end of the task.

For data collection, strawberries were purchased from a supermarket at an average price of 120 JPY per piece. The TDS and TL tasks were conducted in an air-conditioned room at a temperature of approximately 23 °C. Trials were performed at least two hours after the participants’ last meal, either in the morning or afternoon.

### 2.2. TDS and TL Curves Along Normalized Time

Following standardized data processing procedures for the TDS and TL methods [2,13,15,16], we normalized the duration of each TDS and TL task so that 0 and 1 correspond to the moments when the start and stop buttons of the graphical user interface were pressed, respectively.

In the TDS method, the proportion of participants selecting each attribute at any given normalized time is calculated. The smoothed temporal representation of this dominance proportion is referred to as the TDS curve. All attribute curves are plotted on a single graph, with the x-axis representing the normalized time. The sum of the dominance proportions at any time point is 1, except during the early phase immediately after the task begins, when some participants may not select any attributes.

The TL curve represents the time series of the mean liking scores across all participants. In this study, liking was rated on a 9-point scale, with 1 being the lowest value. Similar to the TDS task, immediately after the task begins, no rating button can be selected. During this period, the score was considered 0 in this study.

### 2.3. Example of TDS and TL Curves

**Figure 1:**
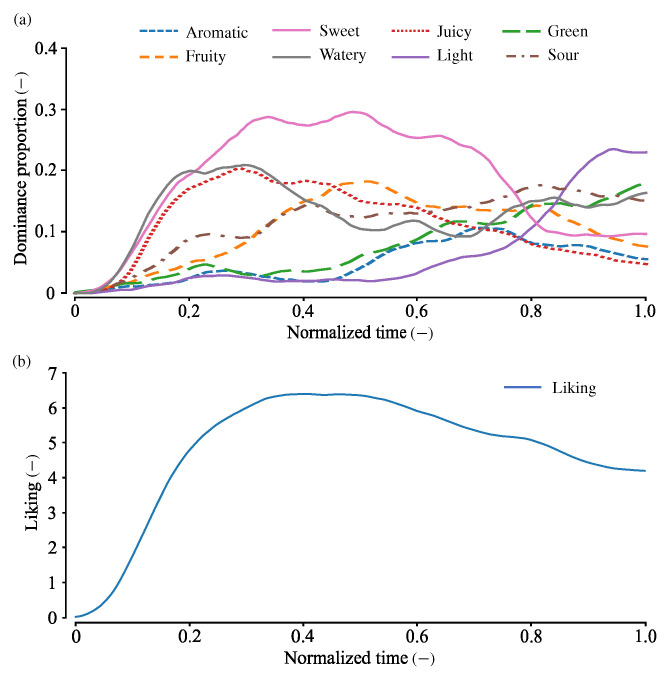
TDS and TL curves for strawberries, calculated from all participants. (**a**) TDS curves. (**b**) TL curve. Adapted from [17].

Figure 1 shows the TDS and TL curves calculated from all the trials (adapted from [17]). In the early phase of tasting, sweet, juicy, and watery sensations were predominant. The sweet sensation remained mostly dominant until approximately 0.8 normalized time. In the last phase, light, green, and sour sensations gradually intensified, while juicy and sweet sensations progressively diminished in dominance.

Temporal liking sharply rose in the early phase of the TL tasks and peaked at approximately 0.3–0.5 normalized time. Then, the liking slightly decreased and ended at approximately 4.

### 2.4. Bootstrap Resampling of TDS and TL Curves and Cross-Validation Methods

In the TDS and TL methods, a single set of curves is typically generated by averaging the data across all participants. However, this is quantitatively insufficient for statistical learning. To address this limitation, bootstrap resampling for time-series sensory appraisal methods [5,8,37] was utilized for data augmentation. This approach enables the generation of multiple TDS and TL curves from a limited sample set while preserving the uncertainty or variation inherent in the original dataset.

We now describe the bootstrap resampling method employed in this study. As outlined in Section 2.1, data from 31 participants were available for both the TDS and TL tasks. These 31 participants were randomly divided into two groups as follows: one used for training and the other for testing. The training set consisted of 16 participants, yielding $N_{1}=48$ samples, while the test set comprised 15 participants, yielding $N_{2}=45$ samples.

For the training data, from the original sample set of size $N_{1}$, a new set of $N_{1}$ samples was randomly drawn with replacement, allowing for duplicates. From this resampled dataset, a set of TDS and TL curves was computed. This process was repeated 100 times in this study, resulting in 100 TDS and TL curve sets. The same protocol was applied to the test data, where a new sample set of size $N_{2}$ was resampled. Thus, a two-fold cross-validation scheme was employed.

By randomly changing the participants included in the training and test sample sets, the resampling procedure was repeated 20 times. Consequently, the two-fold cross-validation process was conducted 20 times in total.

## 3. Time-Series Expansion of Supervised Non-Negative Matrix Factorization

### 3.1. Classification of Matrix Factorization Techniques for Time-Series Data

**Figure 2:**
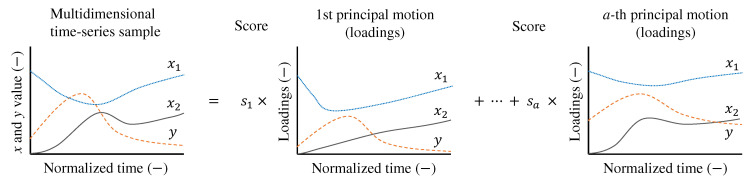
Synergy analysis to decompose the multivariate ($x_{1},\;x_{2},\;y$) time-series sample into a linear combination of multiple principal motions. Principal motions are weighted by scores ($s_{1},\ldots ,s_{a}$). *a* is the number of principal motions.

Matrix factorization techniques for time-series data are linear analysis methods applied to multivariate time-series data. As shown in Figure 2, these methods decompose multivariate time-series data into multiple basis functions, referred to as principal motions in this study. Each principal motion represents a fundamental motion synergy, and any time-series sample can be synthesized from multiple such elements. The principal motions are non-parametric, meaning that they do not assume a predefined functional form, such as sinusoidal or Gaussian functions. This flexibility allows the matrix factorization techniques to adapt to various types of time-series data without imposing restrictive assumptions.

The number of basis functions is determined based on the complexity of the problem domain. For TDS curves, it is typically around three [5]. Including more principal motions allows for a more precise representation of the training data; however, excessive basis functions may lead to overfitting. Therefore, the number of principal motions must be carefully tuned.

In this linear decomposition, the weights assigned to the principal motions are called scores. Once the decomposition is performed, each multivariate time-series sample is projected onto a lower-dimensional basis space, where the coordinates correspond to the scores. A key advantage of linear methods is their interpretability. Both the principal motions and their associated scores are actively analyzed and interpreted.

Synergy analyses based on matrix factorization can be categorized based on the method used to compute the basis functions. First, these methods can be classified into unsupervised and supervised methods. The unsupervised method is, for example, an extension of the principal component analysis, where the basis functions are determined such that the variance of the scores is maximized for each basis. This or the modified methods (e.g., the method based on the factor analysis or independent component analysis) are popular as a method for synergy analysis in biomechanics and neuroscience and are used to investigate human body motions, which represent the spatiotemporal coordination of multiple body parts [25,27,28,29,30,38].

Supervised methods extend partial least squares (PLS) regression to time-series data [32]. PLS is categorized into two types as follows: PLS1, where the dependent variable is a single scalar value; and PLS2, where the dependent variable consists of multiple scalar values or multidimensional data. Both types can be extended to time-series data.

Another classification criterion is the polarity of the following treated signals: unipolar (non-negative) or bipolar. For TDS and TL curves, non-negative versions of the synergy analysis are preferred, as these curves take only non-negative values. These versions decompose time-series data defined by positive values into principal motions and scores that are also strictly non-negative. As a result, the non-negative method enhances the interpretability of principal motions for datasets containing only positive values. The non-negative version of the method, that is, non-negative matrix factorization, has been also applied for synergy analyses [31,39,40,41]. Further, this approach was used for TDS curves of food samples [5], where TDS curves recorded from different food samples were classified without using labeled food categories.

In this study, we focus on supervised non-negative factorization for time-series data, as it enhances the interpretability of principal motions in TDS and TL curves, which contain only positive values. We employ this method to estimate TL curves from TDS curves.

### 3.2. Non-Negative Matrix Factorization for Vector-Valued Targets

Since the supervised non-negative synergy analysis is an application of non-negative matrix factorization to time-series data, we first describe the concept of non-negative matrix factorization.

Let the matrix $X\in R_{0}^{+\;q\times n}$ represent a dataset of *n* samples, each defined by *q* variables that take only non-negative values. Here, $R_{0}^{+}$ denotes the set of non-negative real numbers, including zero. This matrix is decomposed into a basis matrix $P\in R_{0}^{+\;q\times a}$, consisting of *a* principal motions (basis vectors) and a score matrix $S\in R_{0}^{+\;a\times n}$, as follows:

(1) $$\begin{array}{c} X\approx PS. \end{array}$$

The factorization is computed iteratively to minimize the root mean squared error between the matrix elements on both sides [42]. Since exact equality is not guaranteed, this decomposition provides an approximate representation.

In the supervised approach, the decomposition is performed such that the target matrix $Y\in R_{0}^{+\;r\times n}$ can be estimated from X. Here, Y consists of *r*-dimensional target variables. To achieve this, non-negative matrix factorization is applied to an augmented matrix combining X and Y as follows:

(2) $$\begin{array}{c} \left[\begin{array}{c} X\\ Y \end{array}\right]\approx \left[\begin{array}{c} P_{x}\\ P_{y} \end{array}\right]S, \end{array}$$

where $P_{x}\in R_{0}^{+\;q\times a}$ and $P_{y}\in R_{0}^{+\;r\times a}$ are the principal motion matrices corresponding to X and Y, respectively. Here, both X and Y share the same score matrix S as follows:

(3) $$\begin{array}{c} \left\{\begin{array}{c} X\approx P_{x}S,\\ Y\approx P_{y}S. \end{array}\right. \end{array}$$

As a result, the target matrix Y can be estimated as follows:

(4) $$\begin{array}{c} Y\approx P_{y}P_{x}^{+}X, \end{array}$$

where $P_{x}^{+}$ represents the generalized inverse of $P_{x}$.

When estimating the target values for a new sample (sample *i*) that is not included in the training of $P_{x}$ and $P_{y}$, the following equation is used:

(5) $$\begin{array}{c} y_{i}\approx P_{y}P_{x}^{+}x_{i}, \end{array}$$

where $x_{i}\in R_{0}^{+\;q\times 1}$ and $y_{i}\in R_{0}^{+\;r\times 1}$ are the explanatory and target variable vectors, respectively.

For the computation of non-negative matrix factorization, the nnmf function of MATLAB (2023b, MathWorks, Inc., Natick, MA, USA) was used in this study. We used an algorithm called the alternating least squares method [42].

### 3.3. Discretization and Alignment of Multivariate Time-Series Data

To apply non-negative factorization to multivariate time-series data, both the discretization of the time-series data and the alignment of multiple variables are required.

Let the number of explanatory variables be *q*, and consider the *i*-th sample (i=1,⋯,n). The time function of the *j*-th variable (j=1,⋯,q) is denoted as $x_{i,j}\left(t\right)$. Here, in the case of TDS tasks, *q* and t∈[0,1] represent the number of sensory attributes and normalized time, respectively.

This function is sampled at intervals of Δt, yielding the following discrete representation:

(6) $$\begin{array}{c} x_{i,j}\left[k\right]=x_{i,j}\left(\left(k-1\right)\Delta t\right),\;\left(k=1,\cdots ,K\right). \end{array}$$

In this study, the number of discrete points is K=1000 and KΔt=1. Let $x_{i,j}$ be the vector storing the discretized values of $x_{i,j}\left(t\right)$ along the time axis as follows:

(7) $$\begin{array}{c} x_{i,j}={\left(x_{i,j}\left[1\right],\cdots ,x_{i,j}\left[K\right]\right)}^{⊤}. \end{array}$$

These time-series vectors for all explanatory variables are then aligned into a single vector as follows:

(8) $$\begin{array}{c} x_{i}={\left(x_{i,1}^{⊤},\cdots ,x_{i,q}^{⊤}\right)}^{⊤}, \end{array}$$

where the size (length) of $x_{i}$ is K×q. The explanatory variable matrix $X\in R_{0}^{+\;(K\times q)\times n}$ stores these aligned vectors for all *n* samples as follows:

(9) $$\begin{array}{c} X=(x_{1},\cdots ,x_{n}). \end{array}$$

Similarly, the target variable matrix Y is constructed from the time function of the target variable y(t). In this study, the target variable is the temporal liking curve, which is univariate. Thus, the temporal liking curve for the *i*-th sample is discretized as follows:

(10) $$\begin{array}{c} y_{i}\left[k\right]=y_{i}\left(\left(k-1\right)\Delta t\right),\;\left(k=1,\cdots ,K\right). \end{array}$$

The discretized liking curve is then stored in the vector $y_{i}$ as follows:

(11) $$\begin{array}{c} y_{i}={\left(y_{i}\left[1\right],\cdots ,y_{i}\left[K\right]\right)}^{⊤}. \end{array}$$

Finally, the target matrix $Y\in R_{0}^{+K\times n}$ is obtained as follows:

(12) $$\begin{array}{c} Y=(y_{1},\cdots ,y_{n}). \end{array}$$

## 4. Results

We determined the number of principal motions as a=3 to achieve the best performance in terms of the root mean squared error (RMSE) between the predicted and observed TL curves. When a=3, the median RMSE in cross-validation was the smallest at 0.3618 (25th percentile: 0.26; 75th percentile: 0.44). For comparison, the RMSE values for other values of *a* were as follows:

- For a=1, the median RMSE was 0.3887 (25th percentile: 0.28; 75th percentile: 0.44).
- For a=2, the median RMSE was 0.3623 (25th percentile: 0.28; 75th percentile: 0.44).
- For a=4, the median RMSE was 0.3646 (25th percentile: 0.27; 75th percentile: 0.44).
- For a=5, the median RMSE was 0.3679 (25th percentile: 0.27; 75th percentile: 0.44).

**Figure 3:**
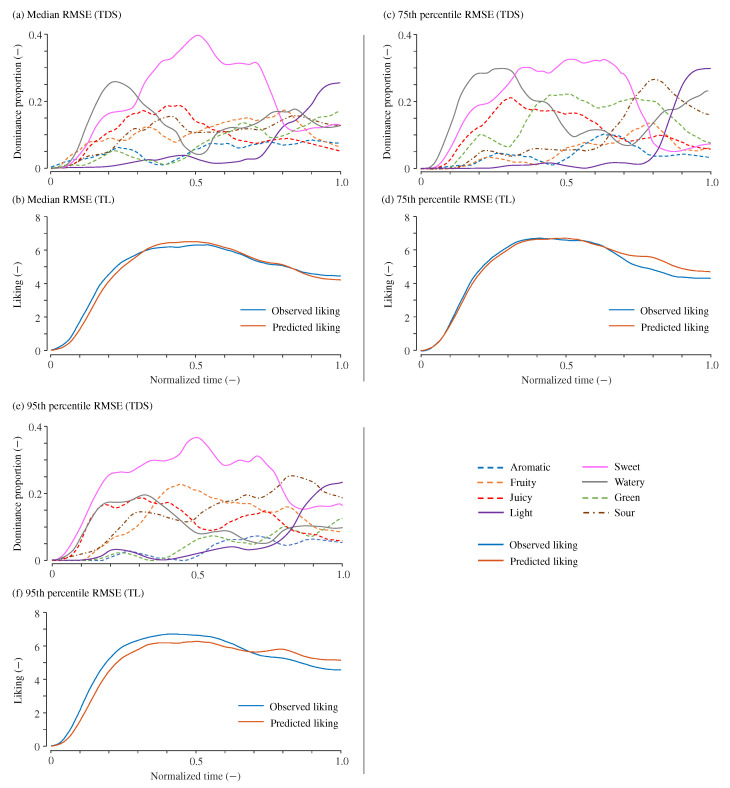
Example of predicted temporal liking curves. (**a**,**b**): Example with a RMSE of 0.36 (median of all tested samples). (**c**,**d**): Example with a RMSE of 0.44 (75th percentile). (**e**,**f**): Example with a RMSE of 0.61 (95th percentile). For each case, the upper panels (**a**,**c**,**e**) display the TDS curves, while the lower panels (**b**,**d**,**f**) show the corresponding observed and predicted TL curves.

Figure 3 presents the observed TDS curves along with the observed and predicted TL curves for samples corresponding to the median (RMSE=0.36), 75th percentile (RMSE=0.44), and 95th percentile (RMSE=0.61) RMSE values when a=3. The figure displays three sets of TDS and TL curves generated through the resampling method, each exhibiting distinct profiles.

**Figure 4:**
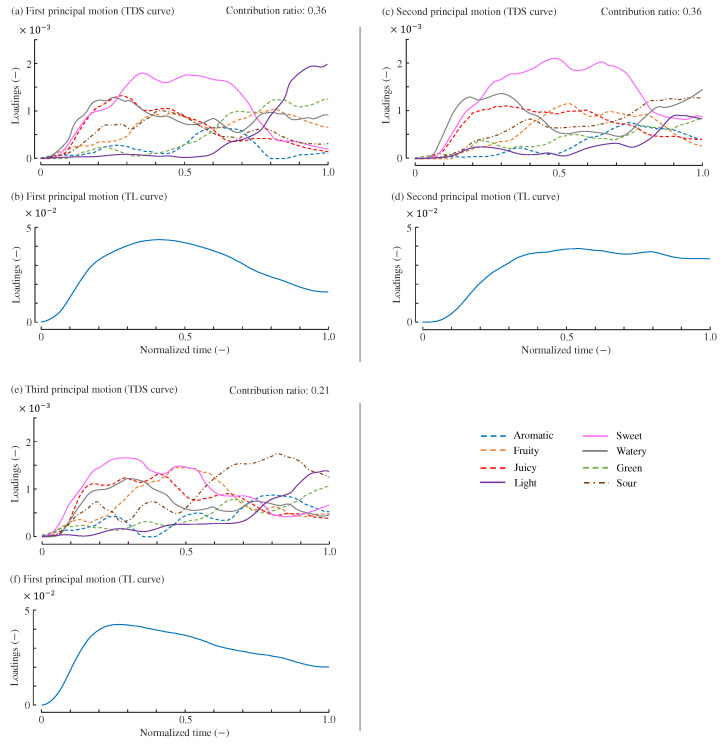
Three principal motions. (**a**,**b**): TDS and TL curves of the first principal motion. (**c**,**d**): TDS and TL curves of the second principal motion. (**e**,**f**): TDS and TL curves of the third principal motion. The vertical axes are expressed in arbitrary units. The contribution ratio of each principal motion is VAF value.

Figure 4 illustrates the shapes of the principal motions when all resampled curves were used for their computation. The figure also includes the Variability Accounted For (VAF) [43,44,45] for each principal motion, which represents their respective contributions to the original data. The VAF values for the three principal motions are 0.36, 0.36, and 0.21, respectively. The VAF values involving the three principal motions was 0.94, indicating that 94% of the variation in the original data was explained using three principal motions. The interpretation of these principal motions is discussed in Section 5.

## 5. Discussion

### 5.1. Interpretation of the Three Principal Motions

Here, we interpret the three principal motions.

The first principal motion, shown in Figure 4a,b, exhibits TDS and TL curves that closely resemble the average curves of strawberries presented in Figure 1. For example, in the early phase of Figure 4a, responses to sweet, juicy, and watery attributes are predominant, with the dominance of juicy and watery gradually decreasing after approximately 0.3 normalized time. In the middle phase, the sweet attribute becomes mostly dominant, while light and green attributes emerge in the final phase. These temporal profiles are similar to those of the average TDS curves. Since only one cultivar of strawberries was analyzed in this study, it is possible that one of the principal motions largely represents the average trend of participants’ responses. Thus, the first principal motion determines the degree of typical sensory and liking responses toward the strawberries tested in [17].

The second principal motion, shown in Figure 4c,d, exhibits distinct TDS and TL curves. In the TDS curve, sour and watery attributes are dominant in the final phase, while green and light attributes are suppressed. In the TL curve, the TL score peaks at approximately 0.50–0.60 normalized time and is generally maintained afterward. These characteristics differ from those of the first principal motion. The suppression of green and light attributes, which are typical of unripe strawberries, in the later phase may explain why the TL score remains high in the final phase. The green and light attributes are known to reduce TL or overall liking for strawberries [17,24,46,47]. Therefore, the second principal motion can be interpreted as representing the characteristics of ripe strawberries.

The third principal motion, shown in Figure 4e,f, is characterized by the prominence of sour and light attributes in the final phase and fruity in the middle phase—features not observed in the other principal motions. Thus, the third principal motion may represent the taste experience of strawberries with pronounced acidity. Notably, the sourness of strawberries is not considered a direct factor in increasing or decreasing liking [17,24,46,47]. The TL curve peaks at 0.20–0.30 normalized time and then gradually declines, with this peak occurring earlier than the average peak in Figure 1.

These principal motions likely reflect individual variations among the tested strawberries or participants. Specifically, the second principal motion represents strawberries with weaker green notes compared to the average, while the third principal motion represents strawberries with stronger sourness. These variations can be attributed to eating methods, individual preferences, and quality differences in the food samples used for sensory evaluation. Strawberries contain relatively higher sugar content at their apex [48], and the way they were masticated in the mouth may have influenced the temporal evolution of taste perception. Furthermore, differences in participants’ preferences may have also contributed to the variation in the curves. Individual differences exist in liking for sourness and sweetness, which are characteristic taste attributes of fruit [49,50,51,52]. Additionally, the strawberries used in the experiment [17] were purchased from supermarkets; thus, cultivation and harvesting conditions were not controlled. This potential variability could be monitored through quality assessment methods, including color analysis [53,54].

### 5.2. General Discussion

In the supervised non-negative factorization method used in this study, the median RMSE for predicting the TL curves of strawberries was 0.36 (25th percentile: 0.26; 75th percentile: 0.44). In a previous study using a reservoir network on the same dataset, the median RMSE was 0.38 (0.29, 0.46) [17]. The estimation accuracy of both methods is comparable.

The reservoir network estimates the current TL value based on both the current and past TDS curves. In contrast, the method employed in this study inherently associates the TL value at a given time point with the entire TDS curve across all time points. This characteristic may not necessarily provide a significant advantage in improving estimation accuracy.

Given that TL values are rated on a 9-point scale, a RMSE of 0.36 is sufficiently small. As shown in Figure 1, even if the maximum average TL value is approximately 6, an error of 0.36 corresponds to about 1/16 of the maximum value. Nonetheless, at present, no formal specifications have been established regarding the required estimation accuracy of TL curves.

The successful estimation of TL curves from TDS curves could reduce the costs associated with sensory evaluation tasks during food development. This cost-reducing effect would be particularly expected when applied to previously untested food products. For instance, Natsume and Okamoto [35] demonstrated a case where the TL curves of a coffee brand were predicted using a machine learning model trained on three other coffee brands. However, it remains unclear whether the method proposed in this study would generalize to such scenarios.

The successful estimation of TL curves from TDS curves would reduce the costs required by sensory evaluation tasks at food development stages. This cost-reducing effect would be especially prominent when the method is applied to non-trained food products. One example was shown in [35], where the TL curves of a coffee brand were predicted by a machine learning model trained by another three coffee brands. It remains unclear whether the method proposed in the present study can adapt to such scenarios.

One important factor influencing estimation accuracy is the selection of sensory attributes used in the TDS method. In the dataset we used, dominant sensory attributes were prioritized, which is appropriate for TDS methods. However, if attributes more strongly related to liking were also considered in the selection process, the accuracy of liking estimation could potentially improve.

The potential improvement in estimation accuracy using machine learning methods, including convolutional neural networks, remains unknown. Some approaches are expected to have a greater generalization capability, allowing a single model to adapt to multiple food products. A key advantage of such methods is their potential applicability to foods for which no data were used in training the estimation model. However, a distinguishing feature of the approach adopted in this study is its interpretability (explainability), which is often difficult to achieve with neural network-based models.

The interpretation of each principal motion is particularly useful when multiple food brands are mixed in the dataset, as it facilitates discussions on differences in curves across brands. As described in Section 5.1, the interpretation of principal motions is often subjective and depends on the researcher’s perspective. To enhance interpretability, non-negative factorization algorithms that enforce sparseness could be employed [55,56,57]. Sparseness refers to a property in which many values in the principal motion loadings are close to zero, which is expected to accentuate differences between principal motions.

In non-negative factorization, the basis matrix (P) and score matrix (S) are initialized with random values, and iterative computations are then performed. Consequently, different principal motions may be obtained in each computation. Although in most cases the resulting principal motions remain similar despite different initial values, they are rarely identical. This inherent characteristic of non-negative factorization could hinder the practical usability of the proposed method, necessitating further investigation to address this issue.

The method presented in this study facilitates the analysis of the interplay between TDS and TL curves, a key aspect of temporal drivers of liking [11,12,13,14,16,17,18,19]. By decomposing these curves into principal motions, which serve as fundamental components of consumers’ sensory and liking experiences, this approach enables a structured examination of their relationships. In the early stages of product development, such analyses could assist in screening and formulation adjustments, providing valuable insights for optimizing sensory attributes.

Although not discussed in this study, an active utilization of principal motion scores may assist in their interpretation. For instance, in the comparison of multiple brands, the correlation between each brand’s compositional content and its principal motion scores could quantitatively define the meaning of the principal motions. In the case of strawberries, numerical attributes, including price, appearance, and chemical and physical properties [58,59], may provide valuable insights when compared with principal motion scores.

While this study demonstrated the applicability of supervised non-negative matrix factorization to analyze TDS and TL curves for strawberries, its generalizability to other food products remains unverified. Although there is no fundamental reason to assume that this method is restricted to strawberries, further validation is required for food products with different sensory dynamics, such as beverages, dairy, and savory foods. Previous studies have successfully applied non-negative matrix factorization to TDS curves of umeboshi (pickled plums) [5], suggesting its broader applicability. Future research should explore its effectiveness across diverse food categories to assess its robustness and potential adaptations.

A future application of this method is its extension to multiple brands. By applying the proposed method to the TDS and TL curves obtained from multiple brands of the same food product, both common and brand-specific principal motions can be extracted. The former suggests temporal drivers of liking that are shared across brands, while the latter indicates brand-specific temporal drivers.

## 6. Conclusions

This study applied supervised non-negative matrix factorization to analyze the synergy between TDS and TL curves. We applied this method on the data collected for strawberries. Our method identified three principal motion synergies accounting for 94% of data variation and predicted TL curves from TDS curves with a median RMSE of 0.36. The extracted principal motions provided interpretable insights into sensory–liking relationships, suggesting key factors influencing liking dynamics in strawberries.

While our approach offers interpretability, its predictive accuracy was comparable to reservoir computing, highlighting the need for further refinement. Additionally, variability in matrix factorization necessitates improvements for reproducibility. Future work should explore sparse factorization techniques and extend this method to multiple food brands to uncover common and brand-specific temporal drivers of liking.

These findings demonstrate the potential of synergy analysis in time-series sensory evaluation, leading to more effective modeling of dynamic taste perception.

## Data Availability

The research data can be accessed by directly requesting access from the corresponding author with a clear explanation of the intended purpose.

## Abbreviations

The following abbreviations are used in this manuscript:

TDS	Temporal Dominance of Sensations
TL	Temporal Liking
PLS	Partial Least Squares
